# Transnasal Flexible Fiberoptic in-office Laryngeal Biopsies—Our Experience with 117 Patients with Suspicious Lesions

**DOI:** 10.5041/RMMJ.10145

**Published:** 2014-04-28

**Authors:** Jacob T. Cohen, Limor Benyamini

**Affiliations:** Voice and Swallowing Disorders Clinic, Department of Otolaryngology Head and Neck Surgery, Rambam Health Care Campus, Haifa, Israel

**Keywords:** Biopsy, in-office, laryngeal cancer, larynx, vocal cords

## Abstract

**Objective:**

To compare pathologic results obtained via in-office transnasal fiberoptic laryngoscopy (TFL) to those of subsequent direct laryngoscopy in order to assess the accuracy of TFL as a diagnostic tool.

**Patients:**

One hundred and seventeen patients with suspicious laryngeal lesions.

**Methods:**

All patients underwent in-office biopsies. All patients with malignant diagnosis were referred to treatment. All patients with benign diagnosis or carcinoma *in situ* were referred to direct laryngoscopy for definitive diagnosis. The pathological results of the specimens from both procedures were compared.

**Results:**

Adequate tissue for diagnostic purposes was obtained in 110 of 117 in-office transnasal fiberoptic laryngoscopy biopsies (94.0%). The biopsy results revealed invasive carcinoma in 42 patients (38.2%), carcinoma *in situ* (CIS) in 17 patients (15.4%), and benign lesions in 51 patients (46.4%). All patients with benign pathologies and carcinoma *in situ* were referred to biopsy under direct laryngoscopy (five patients refused and were removed from the statistics). The final pathologies identified from the biopsies on direct laryngoscopy revealed that there was an underestimation of the transnasal fiberoptic laryngoscopy results in 33 patients (a false negative rate of 31.4%, 33/105) and an overestimation in one patient. The sensitivity of transnasal fiberoptic laryngoscopy biopsy compared with direct laryngoscopy biopsy was 70.6% and the specificity was 96.7%.

**Conclusions:**

TFL with biopsy is easy, safe, and cost-effective but raises serious doubts about its clinical value due to its low sensitivity rate for diagnosing suspicious lesions of the larynx. As such, it is recommended that all patients with a suspicious lesion diagnosed by TFL biopsy as being benign or CIS should be referred to direct laryngoscopy for verification of the findings.

## INTRODUCTION

Laryngeal biopsies have traditionally been done in the operating room under general anesthesia in order to allow access for the cup biopsy into the larynx. Recent advances in technology such as the flexible fiberoptic and the distal chip scope allow these procedures to be performed in awake, unsedated patients. Transnasal fiberoptic laryngoscopy (TFL) has been used to direct various laryngeal procedures, such as the injection of botulinum toxin for the treatment of spasmodic dysphonia,^1^ vocal fold augmentation,^2^ laser manipulations for the treatment of laryngeal dysplasia and papillomatosis,^3^–^7^ removal of benign vocal cord lesions, and laryngeal biopsy.^8^,^9^

Until ∼15 years ago, the primary means for awake laryngopharyngeal biopsy was transoral passage of long curved biopsy forceps with indirect mirror laryngoscopy guidance. With the introduction of the flexible channeled endoscopes and the flexible endoscopes with a channeled sheath, the procedure has become considerably better-tolerated by patients as well as easier to perform. Theoretically these procedures can replace direct laryngoscopy under general anesthesia for the purpose of obtaining tissue for histology. Publications on in-office laryngeal biopsy have concurred that this procedure is safe, feasible, cost-effective, and easy to perform.^8^–^11^ However, only two studies look at the accuracy of in-office biopsy via TFL in patients with strongly suspected laryngopharyngeal cancer. The study from the Boston University Medical Center was a retrospective review on 11 patients that underwent in-office cup forceps biopsies between the years 2006 and 2008. The biopsies taken were only 64% diagnostic.^12^ Our group ran a prospective cohort study on 102 patients and found a 66% agreement between the office-based and the operating room biopsy results.

The sensitivity of TFL biopsy compared with that of direct laryngoscopy biopsy was 69.2%, and the specificity was 96.1%.^13^

This study is a continuation of our previous study with a larger group of patients.

## PATIENTS AND METHODS

All patients who were evaluated in the outpatient clinic and underwent in-office biopsies for suspicious lesions of the larynx were recruited after signing an informed consent. Patients with discrete suspicious-appearing lesions were eligible for inclusion in this study. Suspicious lesionsincluded: leukoplakia, erythroplakia, ulceration, a cauliflower appearance, and a lesion on an immobile vocal cord, thus excluding patients with benign-appearing lesions, such as polyps, nodules, Reinke’s space edema, and findings compatible with chronic laryngitis due to reflux. Patients with suspicious lesions were referred for TFL biopsy in order to determine whether the lesion was malignant or benign.

The pathologic diagnosis of invasive carcinoma from a TFL biopsy was considered equivalent to the pathology results from a direct laryngoscopy biopsy. All patients with benign pathology or carcinoma *in situ* (CIS), however, were referred to subsequent direct laryngoscopy for definitive diagnosis. CIS results were added to invasive carcinoma results when sensitivity and specificity measurements were calculated. Pathological results of the specimens from both procedures were compared.

All relevant demographic and clinical data were retrieved for analysis. The study was approved by the institutional ethics committee, and all suitable patients signed an informed consent form prior to undergoing the procedure.

### Biopsy Technique

We use a Pentax-FNL-10 RP3 (Montvale, NJ, USA) and ENT 2000-vision sciences (Orangeburg, NY, USA) for performing flexible TFL. The endoscope is connected proximally to a camera and monitor. The soft palate is locally anesthetized with 10% xylocaine spray, and the nasal cavity is anesthetized with 2% tetracaine mixed with 0.05% oxymetazoline HCl. The endoscope is covered with a disposable plastic sheath that has a working channel (ENT slide-on Endo-sheath system, Medtronic, Minneapolis, MN, USA). A 2-mm diameter biopsy forceps is inserted through the working channel (Laryngeal Biopsy Forceps, Medtronic, Minneapolis, MN, USA). After insertion of the endoscope, 2 mL of 2% lidocaine is injected through the working channel. In some cases more than one biopsy specimen was collected in order to sample different parts of the lesion. The tissue was collected in a designated pathology plastic cup containing 0.9% NaCl solution. The patients remained for observation in the clinic for 30 minutes after undergoing the procedure.

## RESULTS

A total of 117 patients that underwent in-office biopsies for suspicious-appearing lesions in the larynx participated in the study. The group included 94 males and 23 females with a median age of 66 years (range 30–89 years). The most common presenting symptom was dysphonia (66.6%, *n*= 78). Other symptoms included dysphagia, chronic cough, throat discomfort, and dyspnea. Sixty-six patients (56.4%) had additional co-morbidities, including ischemic heart disease, chronic renal failure, chronic lung disease, and a history of prior cerebrovascular accident; 71 patients (60.6%) were smokers.

Adequate tissue for pathological studies was obtained in 110 of 117 in-office TFL biopsies (94.0%). The other seven patients were referred for further evaluation under direct laryngoscopy, and their data were excluded from the final statistical analysis (in all these cases inadequate tissue was a result of the patients’ intolerance to the procedure).

Fifty-one patients (46.4%, 51/110) had benign pathology, and they were all referred to direct laryngoscopy for subsequent evaluation.

Forty-two patients (38.2%, 42/110) were diagnosed as having invasive carcinoma, and they were all referred directly to definitive treatment (radiotherapy, combined chemo-radiation, and/or surgery) after completing their staging work-up.

Seventeen patients were diagnosed as having carcinoma *in situ* (CIS) (15.4%, 17/110), and they were all referred to direct laryngoscopy in order to confirm the diagnosis, although only 12 patients agreed to do so. All five patients who refused to undergo direct laryngoscopy were referred to the oncology unit, and their data were excluded from final statistical analysis, leaving the data of a total of 105 patients for statistical analysis.

A total of 63 patients (60.0%, 63/105) underwent direct laryngoscopy following TFL: 51 patients with a benign pathology results underwent direct laryngoscopy for subsequent evaluation. Of these, 29 had benign pathology, 18 were diagnosed as having invasive carcinoma, and four had CIS.

Twelve patients with a pathology result of CIS underwent direct laryngoscopy for subsequent evaluation. Of these, biopsies in the operating room revealed 10 cases of invasive carcinoma, one case of CIS, and one case of benign pathology (Table 1).

**Table 1.** Accuracy of Transnasal Flexible Fiberoptic Laryngoscopy.

**DL**	**Benign No.**	**CIS No.**	**SCC No.**	**Total No.**

**TFL**				
Benign	29	4^*^	18^*^	51 (48.7%)
CIS	1^*^	1	10^*^	12 (11.4%)
SCC	0	0	42^†^	42 (40.0%)
Total	30 (28.6%)	5 (4.7%)	70 (66.7%)	105 (100%)

* There was a discrepancy in the TFL and DL pathology results.

† Invasive carcinoma was considered as conclusive pathology results equal to DL pathology results.

Benign, benign TFL pathology result; CIS, carcinoma *in situ*; DL, direct laryngoscopy; SCC, squamous cell carcinoma; TFL, transnasal fiberoptic laryngoscopy.

The final pathologies identified from the biopsies on direct laryngoscopy revealed that there was an underestimation of the TFL results in 32 patients (a false negative rate of 30.4%, 32/105) and an overestimation in one patient (this last-mentioned patient underwent direct laryngoscopy 3 months later due to persistent disease, with the final pathology of the sequential biopsy revealing invasive carcinoma).

In order to calculate the sensitivity and specificity of TFL in the diagnosis of malignant laryngeal lesions, we divided our pathological results into two groups: 1) benign pathology results group, and 2) invasive carcinoma and CIS pathology results group. The sensitivity of TFL biopsies compared with direct laryngoscopy biopsies was 70.6%, and the specificity was 96.7% (Table 2). The positive and negative predictive values in our study were 98% and 57%, respectively.

**Table 2.** Sensitivity and Specificity of Transnasal Fiberoptic Laryngoscopy.^*^

**DL**	**Benign No.**	**CIS/SCC No.**	**Total No.**

**TFL**			
Benign	29	22	51 (48.6%)
CIS/SCC	1	53	54 (51.4%)
Total	30 (28.6%)	75 (71.4%)	105 (100%)

* Sensitivity, 70.6%;Specificity, 96.7%.

Benign, benign TFL pathology result; CIS, carcinoma *in situ*; DL, direct laryngoscopy; SCC, squamous cell carcinoma; TFL, transnasal fiberoptic laryngoscopy.

Complications of in-office TFL were limited to a post-procedure aspiration in one patient (without serious consequences) and a self-limited epistaxis in two patients.

## DISCUSSION

Office-based procedures utilizing new technologies, such as indirect flexible laryngoscopy, are becoming popular, offering a simple, safe, and cost-effective alternative to the traditional direct laryngoscopy procedures, especially for patients who are not candidates for general anesthesia or laryngeal suspension.

The question is whether or not TFL with biopsy gives accurate final pathological results. According to our statistical analysis, the specificity of TFL in diagnosing invasive carcinoma is excellent, but the sensitivity of diagnosing a suspicious lesion as being CIS or invasive carcinoma is only 70.6%. The only other study asking the same question showed 64% diagnostic results in a small group of 11 patients with suspicious laryngeal lesions.^12^ Although in this Boston University study the biopsies were taken using distal chip camera video endoscope, which is superior to our study’s conventional fiberoptic endoscope, our diagnostic results were similar, if not even better (68.6%).

Nearly all other studies on in-office endoscopic biopsies had focused on suspected lesions of the upper aerodigestive tract, and mainly on the esophagus and hypopharynx. Postma et al.^14^ reported 100% accuracy of transnasal esophagoscopy in 17 patients with known lesions of the upper aerodigestive tract. Esophageal biopsies obtained by means of transnasal esophagoscopy are easier to achieve than those from the larynx due to gag and cough reflexes. Thus, improper sample sizes and imprecise biopsies may bias results.

Price et al.^15^ reviewed 18 patients who underwent transnasal flexible laryngo-esophagoscopy for 12 cases of laryngeal lesions. Those authors expressed concern that the size of the acquired biopsy might result in an underestimation of the depth of invasion. Wang et al.^16^ evaluated the efficacy of non-sedated transnasal esophago-gastro-duodenoscopy in the diagnosis of esophageal lesions and reported an 11.1% rate of inaccurate pathological diagnosis among 27 patients with hypopharyngeal cancer. Noteworthily, the conclusions of all the above-mentioned studies were drawn from results derived from much smaller cohorts than the one reported herein and were not compared with biopsies taken under direct laryngoscopy.

It is our impression that pathologists are reluctant to conclude that cancer is present in laryngeal biopsies from small samples. A study by Sarioglu et al.^17^ in which laryngeal pre-neoplastic lesions were evaluated by 14 different pathologists using the World Health Organization, Ljubljana, and squamous intraepithelial neoplasia classification systems concluded that there was a significant difference between the participants in all three classification systems, and the authors questioned intra-observer accuracy.

There is a lack of willingness on the part of the pathologists to commit to a final pathologic diagnosis of CIS/invasive carcinoma based on small fragments of tissue obtained via TFL.

We used fiberoptic equipment in order to achieve the laryngeal view in our current work. Perhaps with improved in-office evaluation with newer distal chip endoscopes and different lighting algorithms (such as narrow band imaging) we would be able to improve our diagnostic yield.

## CONCLUSION

TFL with biopsy is easy, safe, and cost-effective. But, due to its low sensitivity rate for diagnosing suspicious lesion of the larynx, it is recommended that all patients with a suspicious lesion diagnosed by TFL biopsy as being benign or CIS should be referred to direct laryngoscopy for verification of the findings.

## Abbreviations:

CIS: carcinoma *in situ*
TFL: transnasal fiberoptic laryngoscope/laryngoscopy.
